# Relationship between Ambient Temperature and Reasonable Heat Dissipation Coefficient of Mass Concrete Pouring Blocks

**DOI:** 10.3390/ma17102187

**Published:** 2024-05-07

**Authors:** Jiaming Zhang, Hongshi Zhang, Yunpeng Zhao, Wenqiang Xu, Min Su, Jinyu Ge, Sheng Qiang

**Affiliations:** 1 College of Water Conservancy and Hydropower Engineering, Hohai University, Nanjing 210098, China; jiamingz0110@hhu.edu.cn (J.Z.); zhaoyp@hhu.edu.cn (Y.Z.); xwqiang@hhu.edu.cn (W.X.); 2 Materials & Structural Engineering Department, Nanjing Hydraulic Research Institutes, Nanjing 210029, China; w8880600@163.com; 3 Guangzhou River Monitoring Center, Guangzhou 510640, China; redstonez@163.com; 4 Zhejiang Design Institute of Water Conservancy and Hydroelectric Power, Hangzhou 310002, China; waitforcherry@163.com

**Keywords:** mass concrete, surface heat dissipation coefficient, concrete edges, early-age temperature field

## Abstract

In engineering practice, similar surface insulation measures are typically applied to different parts of mass concrete surfaces. However, this can lead to cracking at the edges of the concrete surface or the wastage of insulation materials. In comparison to flat surfaces, the edges of mass concrete structures dissipate heat more rapidly, leading to more pronounced stress concentration phenomena. Therefore, reinforced insulation measures are necessary. To reduce energy consumption and enhance overall insulation effectiveness, it is essential to study the specific insulation requirements of both the flat surfaces and edges of concrete separately and implement targeted surface insulation measures. Taking the bridge abutment planned for pouring in Nanjing City as the research object, this study established a finite element model to explore the effects of different ambient temperatures and different surface heat dissipation coefficients on the early-age temperature and stress fields of different parts of the abutment’s surface. Based on simulation results, reasonable heat dissipation coefficients that meet the requirements for crack prevention on both the structure’s plane and edges under different ambient temperatures were obtained. The results indicate that under the same conditions, the reasonable heat dissipation coefficient at the edges was smaller than that on the flat surfaces, indicating the need for stronger insulation measures at the edges. Finally, mathematical models correlating ambient temperature with reasonable heat dissipation coefficients for the structure’s plane and edges at these temperatures were established, with high data correlation and determination coefficients (R^2^) of 0.95 and 0.92. The mathematical models were validated, and the results from finite element calculations were found to be consistent with those from the mathematical models, validating the accuracy of the mathematical models. The conclusions drawn can provide references for the insulation of similar engineering concrete planes and edges.

## 1. Introduction

After the pouring of mass concrete structures, the rapid hydration heat of cement causes a sharp increase in internal temperature. Meanwhile, the surface of concrete dissipates heat quickly, resulting in significant temperature gradients within the concrete layer and the potential formation of surface tensile stresses. As the concrete reaches its peak temperature, it gradually cools down, leading to volume shrinkage and the generation of considerable tensile stresses under certain constraints [1]. If these tensile stresses exceed the tensile strength of the concrete, internal cracks may occur, even extending to form penetrating cracks, compromising the integrity and durability of the concrete structure. Temperature fluctuations not only induce cracking but also exert significant influences on the stress state of the structure. At times, temperature-induced stresses may numerically surpass those induced by other external loads. Similarly, when pouring new concrete onto existing structures, not only will the temperature-induced stresses be generated, but the expansion of the newly poured concrete will also be constrained by the existing structures [2], further increasing the risk of crack formation.

Therefore, conducting simulation studies on the temperature and stress field of concrete structures during the early-age phase is of paramount practical significance to prevent temperature-related cracking. Many scholars have made significant progress in various aspects such as cement types [3,4,5], concrete mix proportions [6,7,8], admixtures [3,9,10,11,12,13], cooling pipe design [3,14,15,16], curing methods [17,18,19], and finite element model optimization [20,21]. Regarding concrete mix proportions, Barbara, Yunus, and others [22,23] proposed that reducing cement content is an effective method to maintain lower concrete temperatures. Fly ash or slag can substitute part of the cement as cementitious materials, with slower hydration rates and less heat generation. Optimizing aggregate grading or using aggregates with higher heat storage capacity, such as basalt aggregates, can also effectively reduce concrete temperature. When controlling temperature differentials within concrete, factors such as the arrangement of cooling systems, ambient temperature, and pouring temperature need to be considered for their effects on the internal temperature field of concrete [24]. Smolana and others [25,26] discussed some analytical and numerical methods for assessing early-age cracking risks, predicting temperature field, and implementing appropriate protective measures, which contribute to the normal use and durability of concrete structures. Zhang [27] and others demonstrated, through a combination of theoretical, empirical, and numerical simulations, that lowering ambient temperature, reducing the initial temperature of poured concrete, increasing cooling water temperature, or enhancing cooling water flow rate can effectively accelerate concrete cooling and reduce temperature differentials between the interior and exterior of concrete. Nilimaa, J [3,4,5] proposed that preheating the existing structure before pouring concrete blocks is also an effective method for reducing tensile stress and minimizing the risk of structural cracking.

**Figure 1:**
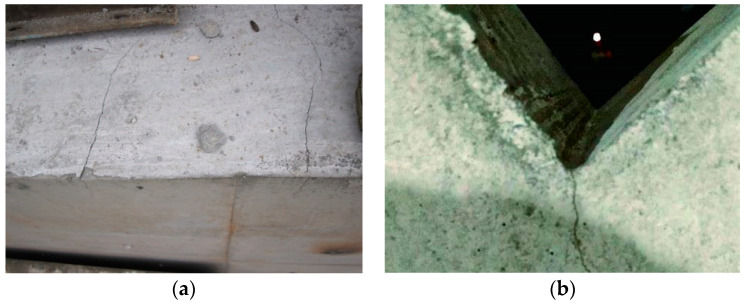
Cracks at the edges of a certain engineering bridge pier. (**a**) Cracks penetrating due to edge cracks; (**b**) Cracks at surface edges.

For mass concrete structures, common surface insulation measures include covering with materials such as expanded polystyrene (EPS) or extruded polystyrene board (XPS), spraying with thermal insulation coatings or polymer coatings, and wrapping with insulation blankets or membranes [28]. Specific measures should be selected based on engineering requirements and environmental conditions [29,30,31]. In engineering practice, the edges of concrete structures are typically considered part of the plane without separately adopting additional insulation measures. However, this practice may increase the risk of structural cracking. Due to their smaller cross-sections and protruding geometric characteristics, edges have higher heat dissipation capacities than flat surfaces. This implies that after concrete pouring, edges dissipate heat more rapidly, leading to quicker temperature reduction and thus increasing temperature differentials, which in turn elevate the likelihood of cracking. The unique geometric shapes of edges also result in more significant stress concentration phenomena, making them more prone to localized high stresses and crack formation. Moreover, due to the limited ductility of concrete materials, once cracks occur at the edges, they are more likely to propagate along the structural depth due to stress concentration effects, leading to more severe structural damage, as illustrated in Figure 1. Therefore, the insulation requirements for the edges of concrete structures are higher than those for flat surfaces. Implementing targeted insulation measures for different parts can maximize the overall insulation effectiveness and reduce energy consumption.

Therefore, this study focuses on a bridge abutment planned for pouring in Nanjing City, investigating the insulation requirements for different surface areas under varying ambient temperatures. The aim is to optimize insulation design to prevent temperature-induced cracks during both the construction and operation phases. The design specification for this abutment requires that the maximum internal temperature of the concrete after casting does not exceed 70 °C, while ensuring that the temperature difference between the surface and the core of the concrete does not exceed 25 °C. In this study, a finite element model was established for a bridge abutment planned for pouring in Nanjing city. Simulation calculations were conducted on its temperature and stress fields under different ambient temperatures and different surface heat dissipation coefficients. Reasonable heat dissipation coefficients satisfying the anti-cracking requirements for both the surface and edges were calculated under different ambient temperatures. A mathematical model correlating ambient temperature with reasonable heat dissipation coefficients for preventing cracking on both the surface and edges was established using the quadratic polynomial fitting method, with correlation coefficients (R^2^) reaching 0.95 and 0.92, respectively, indicating high fitting accuracy. Additionally, simulation calculations were performed on the early-age stress fields of the abutment under ambient temperatures of T = 10 °C and T = 43 °C, validating the accuracy of the mathematical model. The conclusions not only provide guidance for temperature control and crack prevention in this project but also offer references for the insulation of similar engineering concrete surfaces and edges.

## 2. Research Subject Overview

The research subject of this study is a bridge pier soon to be constructed in Nanjing city. The main bridge has a total length of 3.67 km and adopts continuous steel box girders with standard spans ranging from 27 to 35 m. The substructure consists of double-column piers or double-column upright piers, with additional auxiliary piers at widened sections, and the foundation is composed of bored pile foundations. The concrete strength grade for the bridge piers and abutments is C30. The dimensions of the abutment structure are 8.9 m (length) by 6.9 m (width) by 2.7 m (height), while those of the bridge piers are 2.0 m (length) by 1.8 m (width), with varying pier heights at different locations, and the base of the abutment is founded on pile foundations. Concrete pouring consists of three layers: the cushion layer, the abutment, and the bridge pier, all poured in one go. The design requirement stipulates that the internal maximum temperature after concrete molding should not exceed 70 °C, while ensuring that the temperature difference between the surface and the core of the concrete does not exceed 25 °C. The abutment of this elevated main bridge falls under the category of large-volume concrete structures and is subject to considerable construction-phase stresses induced by factors such as self-weight, temperature, autogenous volume deformation, and creep. Among these, the construction-phase stresses induced by temperature, autogenous volume deformation, and creep are difficult for designers and specifications to fully consider. However, in reality, for large-volume structures under significant constraints, these stresses often exhibit considerable magnitudes. Concrete’s weakness lies in its low tensile strength, making it prone to cracking under tensile stress during construction phases. With age, tensile stresses accumulate, leading to greater tensile stresses during operation, posing greater risks to the integrity and durability of the structure. Therefore, conducting finite element calculations on the temperature and stress fields of the abutment under different conditions is of significant practical importance for predicting potential cracks during the construction and operation phases.

## 3. Basic Calculation Principles

### 3.1. Basic Principles of Unsteady Temperature Field Finite Element

At any point within the concrete computational domain (*R*), the unsteady temperature field (*T*(*x*, *y*, *z*, *t*)) must satisfy the following heat conduction control equation [32]:

(1) $$\frac{\partial T}{\partial t}=a\left(\frac{\partial^{2}T}{\partial x^{2}}+\frac{\partial^{2}T}{\partial y^{2}}+\frac{\partial^{2}T}{\partial z^{2}}\right)+\frac{\partial \theta }{\partial \tau }(\forall (x,y,z)\in R)$$

where *T* is the temperature (°C); *α* is the thermal conductivity coefficient (m²/h); *θ* is the adiabatic temperature rise for concrete (°C); *t* is the time (days); *τ* is the age (days).

For Equation (1), a unique solution is obtained by introducing corresponding initial and boundary conditions. The expression for the initial condition is given by Equation (2).

(2) $$T=T(x,y,z,t_{0})$$

The boundaries of the concrete structure’s temperature field calculation domain (*R*) are typically divided into three types. The first type of boundary condition comprises known temperature boundaries, including the initial temperature of the concrete upon pouring and the initial temperature of the bedrock. The second type of boundary condition is adiabatic. The third type of boundary condition is surface heat exchange [33]. Their expressions are as follows:

(3) $$\left\{ \begin{array}{l} T(x,y,z,t)=f(x,y,z,t)\\ \frac{\partial T(x,y,z,t)}{\partial n}=0\\ -\lambda \frac{\partial T(x,y,z,t)}{\partial n}=\beta (T(x,y,z,t)-T_{a}(x,y,z,t)) \end{array}\right.$$

where *β* is the heat release coefficient (kJ/(m^2^·h·°C)); *λ* is the thermal conductivity coefficient (kJ/(m·h·°C)); *T_a_* is the ambient temperature (°C).

### 3.2. Finite Element Method Calculation for Creep Stress

Zhu [33] provides a detailed description of the specific calculation process. The strain increment for concrete under complex stress conditions often includes elastic strain increment, creep strain increment, temperature strain increment, drying shrinkage strain increment, and autogenous volume deformation increment [34,35]; thus,

(4) $$\left\{\Delta \varepsilon_{n}\right\}=\left\{\Delta \varepsilon_{n}^{e}\right\}+\left\{\Delta \varepsilon_{n}^{c}\right\}+\left\{\Delta \varepsilon_{n}^{T}\right\}+\left\{\Delta \varepsilon_{n}^{S}\right\}+\left\{\varepsilon_{n}^{0}\right\}$$

where $\left\{\Delta \varepsilon_{n}^{e}\right\}$ is the elastic strain increment; $\left\{\Delta \varepsilon_{n}^{c}\right\}$ is the creep strain increment; $\left\{\Delta \varepsilon_{n}^{T}\right\}$ is the temperature strain increment; $\left\{\Delta \varepsilon_{n}^{S}\right\}$ is the drying shrinkage strain increment; $\left\{\varepsilon_{n}^{0}\right\}$ is the autogenous volume deformation increment.

## 4. Simulation of Temperature Field and Stress Field

### 4.1. Calculation Parameters

#### 4.1.1. Air Temperature Parameters

The location of the project is in Nanjing, Jiangsu Province, China. The average monthly air temperatures in the area over several years are shown in Table 1. For computational convenience, the average monthly air temperatures are fitted to a cosine curve, expressed by the following formula:

(5) $$T_{a}(t)=16.5+11.7\times \cos\left[\frac{\pi }{6}\left(t-7.3\right)\right]$$

The daily variation curve is expressed by the following formula:

(6) $$T_{a}^{d}(t)=T_{a}+6\times \cos\left[\frac{\pi }{12}\left(t-14\right)\right]$$

where *t* is the time within each day (hours). *T_a_* is the daily average air temperature.

#### 4.1.2. Thermal and Mechanical Parameters of Foundation and Concrete

The abutment design adopts C30 commercial concrete for pouring. Based on relevant specifications, on-site experiments, and the geological report of this project, the thermal and mechanical parameters of the foundation layer and concrete are determined, as shown in Table 2.

**Figure 2:**
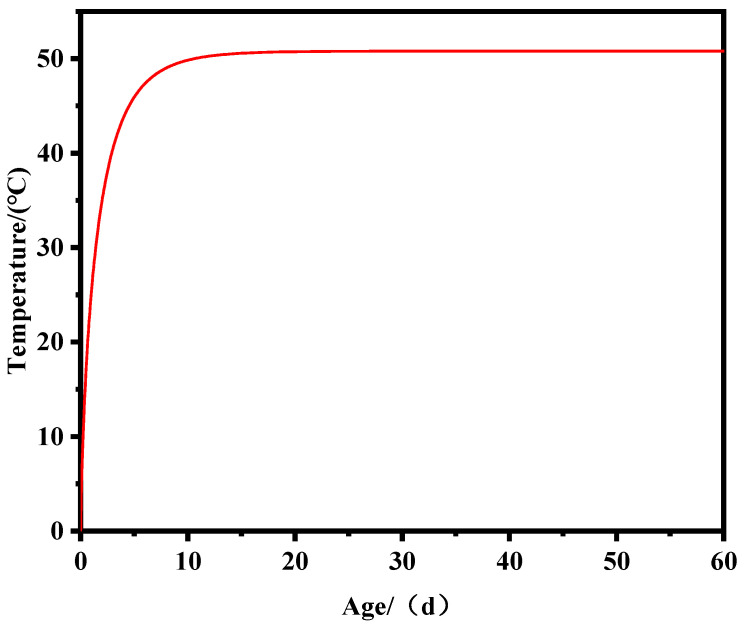
Adiabatic temperature rise duration curve.

Through concrete adiabatic temperature rise tests, the temperature variation curve of concrete was measured, as shown in Figure 2. In the calculation of concrete temperature field in this study, the adiabatic temperature rise is modeled using the composite exponential model proposed by Zhu [32], and the expression is fitted as shown in Equation (7).

(7) $$\theta (\tau )=50.8\times (1-e^{-0.69\tau^{0.76}})$$

### 4.2. Finite Element Model

**Figure 3:**
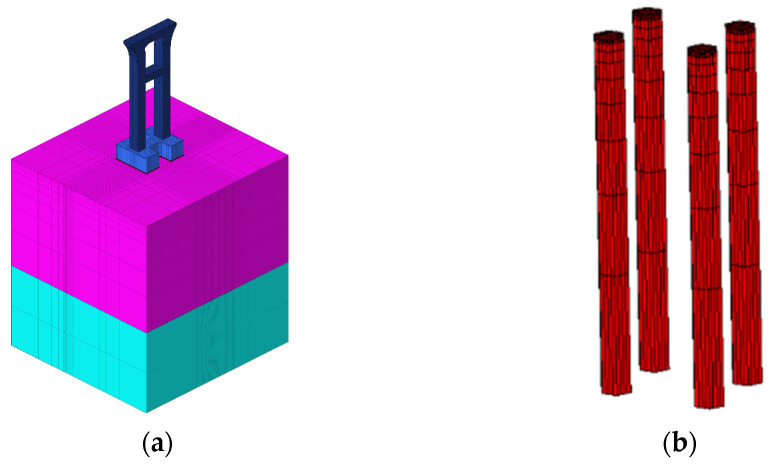

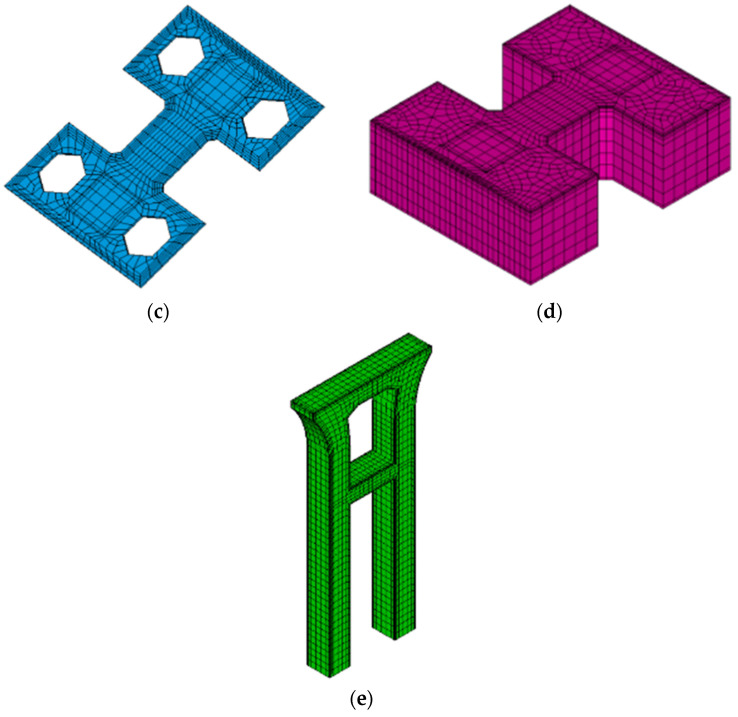
Finite element model. (**a**) Overall finite element model; (**b**) concrete pile finite element model; (**c**) cushion finite element model; (**d**) abutment finite element model; (**e**) pier finite element model.

By modeling and simulating the structure with reasonable boundary conditions, the modeling objects include the cushion, abutment, and pier. In this study, only the temperature field and stress field calculation results of the abutment are analyzed. The finite element models are shown in Figure 3, with a total of 35,752 elements and 40,834 nodes.

### 4.3. Calculation Conditions

In the temperature field simulation calculation, the surroundings and bottom surface of the foundation are adiabatic surfaces, and the top surface is a heat dissipation boundary. The exposed surfaces of the concrete structure are all heat dissipation boundaries. In the stress field simulation calculation, the surroundings and bottom surface of the foundation are subjected to normal constraints, and the top surface is a free boundary. Other surfaces are all free boundaries.

**Figure 4:**
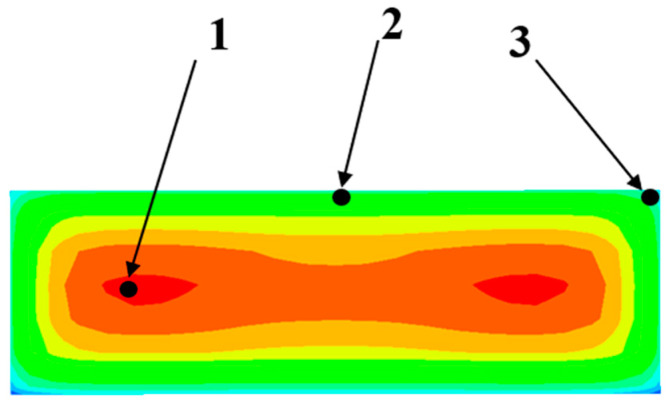
Positions of key points.

To study the influence of surface heat dissipation coefficients on the early-age temperature field and stress field of different parts of the structure under different ambient temperatures, this study selects a key point inside the structure (Point 1), one on the surface plane (Point 2), and another on the edges (Point 3) for analysis, as shown in Figure 4. According to temperature data, the monthly average temperatures for April, October, May, and July are 14.3 °C, 18.3 °C, 21.7 °C, and 28.8 °C, respectively, showing a relatively uniform gradient. Therefore, this study uses the aforementioned monthly average temperatures as different ambient temperatures for research. Additionally, conditions with ambient temperatures of T = 34 °C and T = 38 °C are set to explore the variations in the early-age temperature field and stress field of the structure under high-temperature conditions during summer construction.

The surface heat dissipation coefficient of concrete depends on the surface covering conditions and wind speed at different stages of construction at the engineering site. The average wind speed in the construction area of this project is 2 m/s. According to engineering experience, when there are no insulation measures, the surface heat dissipation coefficient β is taken as 48 kJ/(m^2^·h·°C). Additionally, conditions with surface heat dissipation coefficients of 40, 32, 25, 16, and 8 kJ/(m^2^·h·°C) are set for comparative studies.

## 5. Results and Discussion

### 5.1. Exploration of the Relationship between Ambient Temperature and Surface Heat Dissipation Coefficient

Compared to the plane of large-volume concrete structures, the edges have a larger contact area with the surrounding environment, making it easier for heat generated by cement hydration to dissipate from the edges, resulting in a larger temperature difference between the interior and exterior. Additionally, the geometric structure of the edges themselves leads to a more significant stress concentration phenomenon, making the edges more prone to early-age cracking relative to the plane. Therefore, stronger insulation measures are needed at the edges. To reduce the risk of structural cracking and optimize the insulation design, this section explores the reasonable heat dissipation coefficients for both the plane and edges of concrete structures under different ambient temperatures.

Standards such as GB/T 51028-2015 “Technical code for temperature measurement and control of mass concrete” typically stipulate that the temperature difference between the interior and exterior of mass concrete structures should not exceed 25 °C [36]. Therefore, in this section, the surface heat dissipation coefficient corresponding to a temperature difference of 25 °C between the interior and surface of the structure is considered as the reasonable heat dissipation coefficient for the plane. Due to the unique geometric characteristics of the edges and their larger contact area with the external environment, the risk of cracking at the edges is more influenced by the internal stress distribution of the structure itself. Hence, the surface heat dissipation coefficient corresponding to tensile stress exceeding the standard value (the difference between tensile stress and tensile strength) of 0 MPa is considered as the reasonable heat dissipation coefficient for the edges.

**Figure 5:**
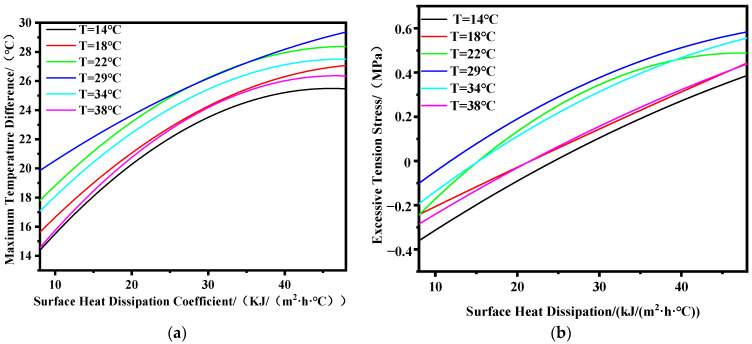
Comparison of calculated results. (**a**) Plane; (**b**) edge.

In this section, the maximum temperature difference between the interior and surface of the structure is obtained by comparing the early-age temperature duration curves of internal key point 1 and surface key point 2 under different combinations of ambient temperatures and surface heat dissipation coefficients. Similarly, the maximum overstress value at the edges is obtained by comparing the early-age stress duration curve of edge key point 3 with the development curve of concrete tensile strength. A mathematical model is established for the results using nonlinear fitting methods, as shown in Figure 5. The fitting curve has very small errors, with a maximum error of 5.41%, a minimum error of 1.67%, and an average error of 3.27%.

**Figure 6:**
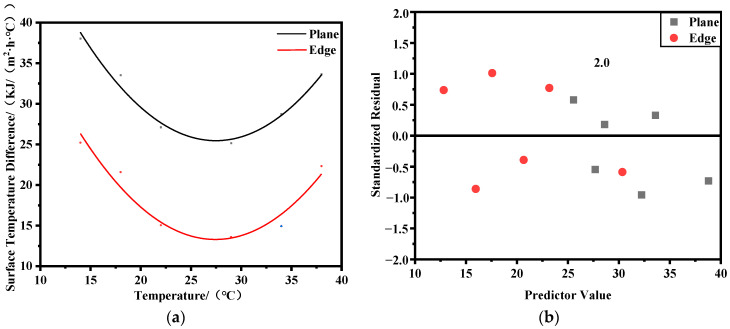
Relationship between ambient temperature and reasonable heat dissipation coefficients. (**a**) Fitting results; (**b**) standardized residual.

During the above calculation process, under the working conditions of ambient temperatures T = 34 °C and T = 38 °C, the peak internal temperature of the structure without water cooling exceeds 70 °C, which does not meet the design requirements. Therefore, a water cooling scheme is adopted to reduce the peak internal temperature. From Figure 5a, it can be observed that as the surface heat dissipation coefficient decreases, indicating the strengthening of insulation measures, both the maximum temperature difference between the interior and exterior and the excessive tensile stress decrease, consistent with the conclusions of Lin’s study [37]. Based on the curves in Figure 5, reasonable heat dissipation coefficients corresponding to a temperature difference of 25 °C between the interior and exterior, as well as an overstress value of 0 MPa at the edges, are obtained under different ambient temperatures. Mathematical models are established for the relationship between ambient temperature and reasonable heat dissipation coefficients for the plane and edges, revealing a parabolic distribution pattern between reasonable heat dissipation coefficients and ambient temperature. The fitting curves are shown in Figure 6, and the expressions are given in Equation (8). The correlation coefficients R^2^ for the fitting curves of the plane and edges reach 0.95 and 0.92, respectively, indicating high fitting accuracy. Figure 6b shows the standardized residual graph, with the x-axis representing the predictor values of reasonable heat dissipation coefficients and the y-axis representing the standardized residual values. From Figure 6b, it can be observed that the standardized residuals are distributed around zero, exhibiting a basic symmetrical distribution. The distribution characteristics remain unchanged with an increase in predictor values, indicating conformity with the assumptions of homoscedasticity and independence of data. Moreover, all standardized residuals fall within the range of −2 to 2, indicating that the error terms follow a normal distribution, suggesting a good model fit.

The corresponding heat dissipation coefficients shown in Figure 6 under specific temperature conditions represent the maximum heat dissipation coefficients that ensure the plane and edges do not crack. If the actual heat dissipation coefficient exceeds the corresponding value on the curve in Figure 6 under the corresponding temperature conditions, there is a risk of cracking on the structure’s surface, and insulation measures should be strengthened. Additionally, it can be observed that the reasonable heat dissipation coefficient to prevent cracking at the edges is smaller than that for the plane, indicating that stronger insulation measures are required at the edges to meet the anti-cracking requirements.

(8) $$\left\{ \begin{array}{l} y_{1}=0.076x^{2}-4.177x+82.638\\ y_{2}=0.072x^{2}-3.962x+67.649 \end{array}\right.$$

In the equation, *x* represents the ambient temperature in °C, *y*_1_ represents the reasonable heat dissipation coefficient for the plane, and *y*_2_ represents the reasonable heat dissipation coefficient for the edges.

### 5.2. Model Validation

**Figure 7:**
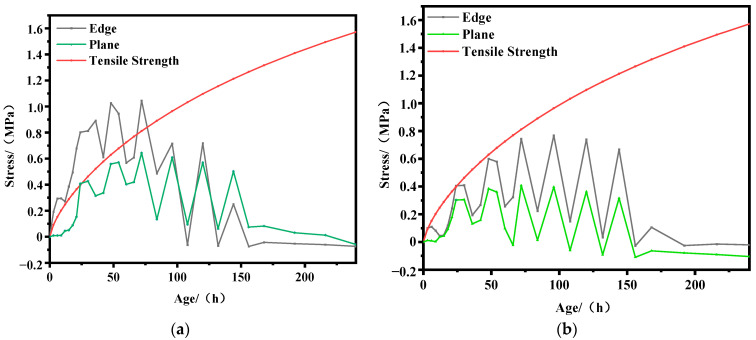
Stress history curves at ambient temperature T = 10 °C. (**a**) β = 48.47 kJ/(m^2^·h·°C); (**b**) β = 35.22 kJ/(m^2^·h·°C).

**Figure 8:**
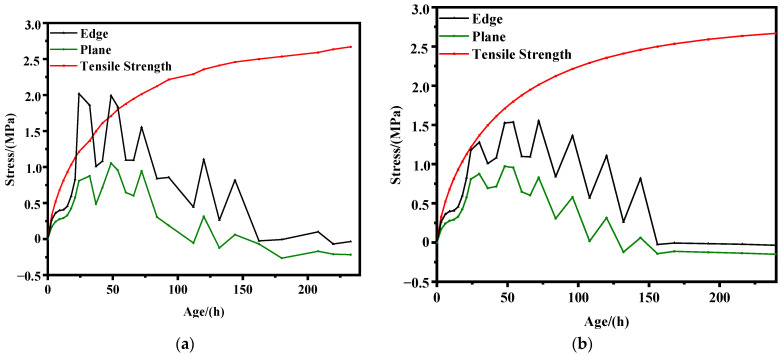
Stress history curves at ambient temperature T = 43 °C. (**a**) β = 43.55 KJ/(m^2^·h·°C); (**b**) β = 32.68 KJ/(m^2^·h °C).

According to Equation (7), the reasonable heat dissipation coefficients for the plane and edges corresponding to an ambient temperature of T = 10 °C are calculated to be 48.47 and 35.22 kJ/(m^2^·h·°C), respectively, and for T = 43 °C, the corresponding values are 43.55 and 32.68 kJ/(m^2^·h·°C). In this section, the early-age stress fields of the structural surface key point 2 and edge key point 3 are analyzed and evaluated under ambient temperatures of 10 °C and 43 °C, respectively, to validate the accuracy of Equation (7). The results are shown in Figure 7 and Figure 8.

From Figure 7a and Figure 8a, it can be observed that when the surface heat dissipation coefficient is set to the same value as the rational surface heat dissipation coefficient, the tensile stress at the edge significantly exceeds the tensile strength within the age range of 0 to 54 h. Additionally, the maximum exceedance of tensile strength at temperatures of T = 10 °C and T = 43 °C can reach 0.70 MPa and 0.40 MPa, respectively. This indicates a high risk of cracking at the edge during the early age. Meanwhile, the tensile stress at the structural surface remains below the tensile strength. Thus, setting the surface heat dissipation coefficient to the rational surface heat dissipation coefficient can meet the requirement of no cracking at the surface, but cracking may still occur at the edge. If further reinforcement measures are taken to adjust the surface heat dissipation coefficient to the rational edge heat dissipation coefficient, the early-age stress history at the structural surface and edge is shown in Figure 7b and Figure 8b. It can be observed that under both environmental temperatures, the tensile stress at the surface remains below the tensile strength, meeting the anti-cracking requirements. At T = 10 °C, the tensile stress at the edge only exceeds the tensile strength at very few moments, and the magnitude of exceedance is very small. At T = 43 °C, the tensile stress at the edge remains below the tensile strength. This indicates that adjusting the surface heat dissipation coefficient to the rational edge heat dissipation coefficient eliminates the risk of cracking at both the edge and surface, thereby verifying the accuracy of Equation (7).

## 6. Conclusions

(1) Based on the finite element analysis of the bridge pier in this project, it can be affirmed that the edges of the structure are more prone to cracking compared to the surface. Stronger insulation measures are needed, and there is a difference of 10~20 kJ/(m^2^·h·°C) in the rational heat dissipation coefficient between the surface and the edge of the pier. By analyzing the finite element calculation results of the pier under different environmental temperatures and surface heat dissipation coefficient conditions, a mathematical model for determining the rational heat dissipation coefficient that prevents cracking on the surface and edge of the pier is established. The coefficient of determination R^2^ for the fitting is 0.95 and 0.92, indicating high fitting accuracy. The fitting curve shows that around an environmental temperature of T = 28 °C, the rational heat dissipation coefficient for both the surface and the edge of the pier is minimal. This indicates a high risk of surface cracking due to the large temperature difference between the inside and outside of the pier at this temperature. Therefore, it is essential to ensure that the heat dissipation coefficient does not exceed the rational heat dissipation coefficient corresponding to the environmental temperature on the fitting curve to prevent surface cracking of the pier.

(2) Applying the relationship between environmental temperature and the rational heat dissipation coefficient proposed in this paper, the rational heat dissipation coefficients for the surface and edge of the pier are calculated at environmental temperatures of T = 10 °C and T = 43 °C. The early-age tensile stress on the surface and edge of the pier under corresponding conditions is compared to verify the accuracy of the formula. The results show good agreement between the finite element analysis results and the mathematical calculation results. The research findings can provide references for the insulation of similar concrete structures’ surfaces and edges. However, it should be emphasized that the research results from Figure 6 are not universally applicable to all ambient temperatures. In extreme low-temperature conditions during winter, additional insulation measures need to be reinforced based on the findings of this study to prevent concrete freezing and expansion.

## Figures and Tables

**Table 1:** Average monthly air temperatures in the local area.

Month	1	2	3	4	5	6	7	8	9	10	11	12
Measured Values (°C)	4	8	10.5	14.6	22	25.3	29.2	28	23	17.5	12.5	4.2
Fitted Values (°C)	4.9	5.6	10.0	14.3	21.7	25.9	28.8	27.4	23.9	18.3	12.3	7.4

**Table 2:** Thermal and mechanical parameters of foundation.

Material	Thermal Conductivity (kJ/(m·h·°C))	Specific Heat (kJ/(kg·°C))	Thermal Diffusivity (m^2^/h)	Linear Expansion Coefficient (10^−6^/°C)	Poisson’s Ratio	Density (kg/m^3^)	Young’s Modulus (GPa)
Foundation	2.31	2.19	0.000583	8.0	0.35	1855	0.01
C30 Concrete	10.44	1.05	0.004400	9.5	0.167	2318	30

## Data Availability

Data are contained within the article.
